# A Mobile Health Coaching Intervention for Controlling Hypertension: Single-Arm Pilot Pre-Post Study

**DOI:** 10.2196/13989

**Published:** 2020-05-07

**Authors:** Himali Weerahandi, Soaptarshi Paul, Lisa M Quintiliani, Sara Chokshi, Devin M Mann

**Affiliations:** 1 Division of General Internal Medicine and Clinical Innovation Department of Medicine NYU Grossman School of Medicine New York, NY United States; 2 Department of Population Health NYU Grossman School of Medicine New York, NY United States; 3 Section of General Internal Medicine Department of Medicine Boston University School of Medicine Boston, MA United States; 4 Department of Medicine NYU Grossman School of Medicine New York, NY United States; 5 Medical Center Information Technology NYU Langone Health New York, NY United States

**Keywords:** eHealth, mobile, telemedicine

## Abstract

**Background:**

The seminal Dietary Approaches to Stopping Hypertension (DASH) study demonstrated the effectiveness of diet to control hypertension; however, the effective implementation and dissemination of its principles have been limited.

**Objective:**

This study aimed to determine the feasibility and effectiveness of a DASH mobile health intervention. We hypothesized that combining Bluetooth-enabled data collection, social networks, and a human coach with a smartphone DASH app (DASH Mobile) would be an effective medium for the delivery of the DASH program.

**Methods:**

We conducted a single-arm pilot study from August 2015 through August 2016, using a pre-post evaluation design to evaluate the feasibility and preliminary effectiveness of a smartphone version of DASH that incorporated a human health coach. Participants were recruited both online and offline.

**Results:**

A total of 17 patients participated in this study; they had a mean age of 59 years (SD 6) and 10 (60%) were women. Participants were engaged with the app; in the 120 days of the study, the mean number of logged blood pressure measurements was 63 (SD 46), the mean number of recorded weight measurements was 52 (SD 45), and participants recorded a mean of 55 step counts (SD 36). Coaching phone calls had a high completion rate (74/102, 73%). The mean number of servings documented per patient for the dietary assessment was 709 (SD 541), and patients set a mean number of 5 (SD 2) goals. Mean systolic and diastolic blood pressure, heart rate, weight, body mass index, and step count did not significantly change over time (*P*>.10 for all parameters).

**Conclusions:**

In this pilot study, we found that participants were engaged with an interactive mobile app that promoted healthy behaviors to treat hypertension. We did not find a difference in the physiological outcomes, but were underpowered to identify such changes.

## Introduction

By 2030, hypertension is expected to affect 41.4% of American adults [1]. However, blood pressure remains insufficiently controlled in almost half of patient cases [2]. The enormous burden of hypertension creates a need for effective interventions with minimal patient burden to encourage successful behavior change. The seminal Dietary Approaches to Stopping Hypertension (DASH) study, published 20 years ago, demonstrated effective control of hypertension through diet [3]. Since then, DASH has been implemented in clinical settings with limited success, partly due to the original intervention design that required substantial in-person participation [4].

DASH for Health, a web-based version of the program, was developed in 2008 to combine the success of the DASH dietary approach of controlling hypertension with evidence-based lifestyle changes (eg, physical activity) and novel internet delivery, thereby reducing the burden of DASH’s original in-person design [5]. DASH for Health has been shown to be effective in hypertension management for those who stay engaged with the program for an extended period [5]; however, web-based interventions are still affected by severe drops in patient utilization after the initial weeks of intervention participation [6]. Mobile versions of DASH have been developed but relied on self-reporting for measures of blood pressure, weight, and physical activity, which increase patient burden and may not be accurate [7]. To overcome these limitations, we developed DASH Mobile, a mobile platform that leveraged smartphone technology with Bluetooth-enabled devices, along with human coaching, to facilitate behavior changes.

The widespread use of smartphones among all sociodemographic groups presents a disruptive opportunity to deliver a more accessible version of DASH while increasing patient adherence to the program [8]. By automatically collecting data outside of the clinic and delivering real-time, personalized messaging to each user, smartphone technology minimizes patient burden while creating a positive feedback loop for behavior change.

Our strategy was to adapt DASH to a smartphone-based platform (DASH Mobile) that leveraged automatic data collection with behavior change support from a human coach to replicate the success of DASH in a less burdensome digital context. The purpose of this study was to evaluate the feasibility and potential clinical effectiveness of DASH Mobile, including its effects on engagement (use and acceptability), physiological (blood pressure), and behavioral (diet and physical activity) outcomes.

## Methods

### Study Design

This was a single-arm pilot study conducted from August 2015 through August 2016 with a pre-post evaluation design to evaluate the feasibility and preliminary effectiveness of a smartphone version of DASH that incorporated health coaching. This study was approved by the Boston University Medical Campus Institutional Review Board.

### Recruitment and Participants

Participants were recruited from the greater Boston area via recruitment materials in the local paper and research registry, using an opt-in paradigm. In addition, emails with information about the study were sent to those deemed eligible from Boston University’s research volunteer registry. Participants were eligible if they were aged 18-65 years, owned an iOS or Android smartphone with a data plan, were English speaking, were currently taking hypertension medication, or had a diagnosis of prehypertension or Stage 1 hypertension, and were able to give informed consent. Prehypertension was defined as a systolic blood pressure of 120-139 mm Hg, and Stage 1 hypertension was defined as a systolic blood pressure of 150-160 mm Hg for adults aged ≥60 years, 140-160 mm Hg for patients with diabetes or chronic kidney disease, or 140-160 mm Hg for all others.

Participants were excluded if they were pregnant or nursing, held a terminal diagnosis, had a diagnosis of secondary hypertension, were unable to easily navigate apps on their smartphone; their baseline blood pressure was in the normal range without blood pressure medication; or they had any contraindications to physical activity. We also excluded patients with medical conditions including dementia, active cancer, or anorexia.

A research assistant obtained informed consent, including HIPPA authorization, from interested participants. The mobile app was then loaded onto the participant’s smartphone, and behavior-tracking devices (pedometer, scale, blood pressure cuff) were synced.

### Intervention

**Figure 1:**
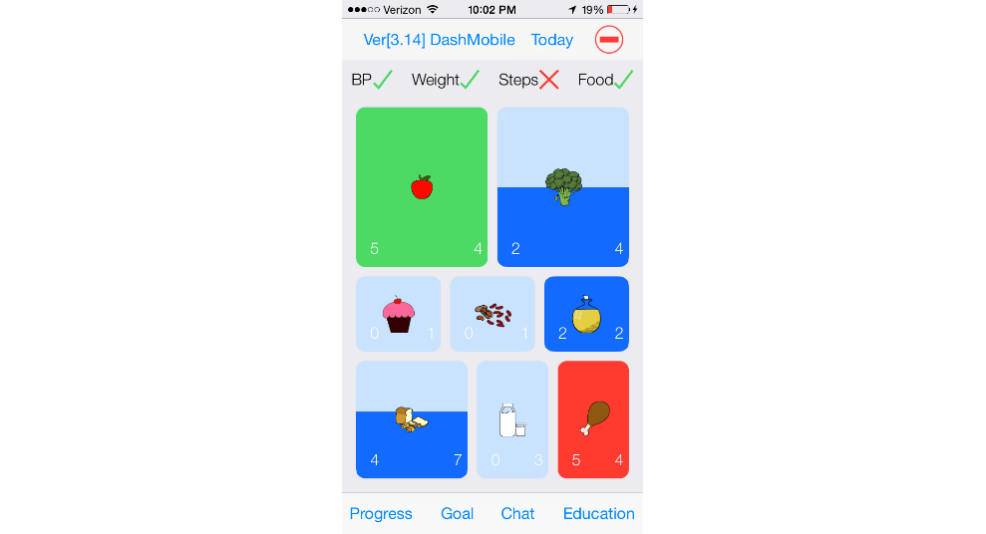
Simplified data entry tool based on the DASH portions-based approach to dietary intake.

**Figure 2:**
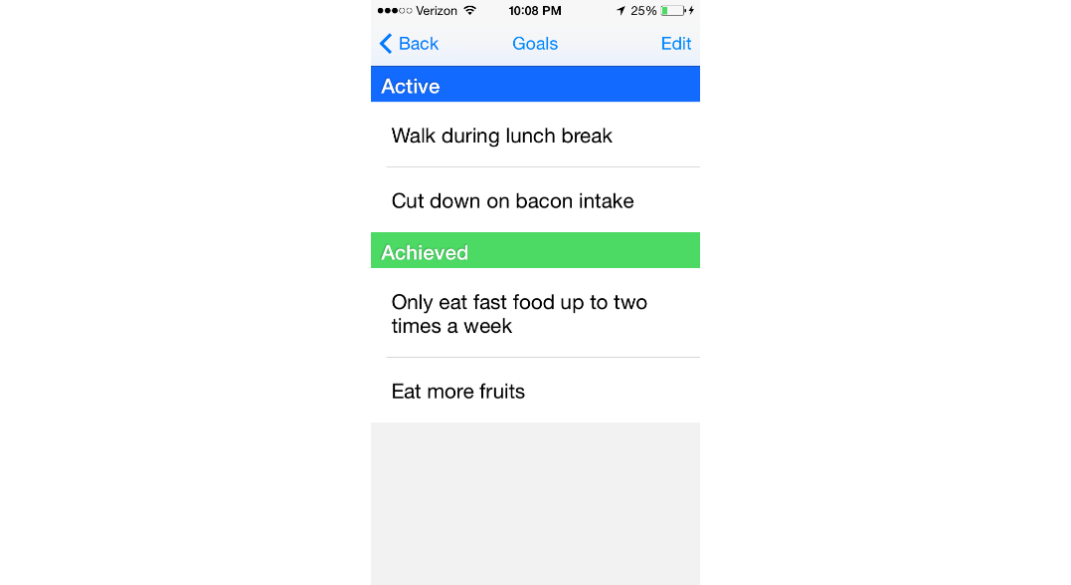
Editable active and achieved goals are listed in the Goals window.

**Figure 3:**
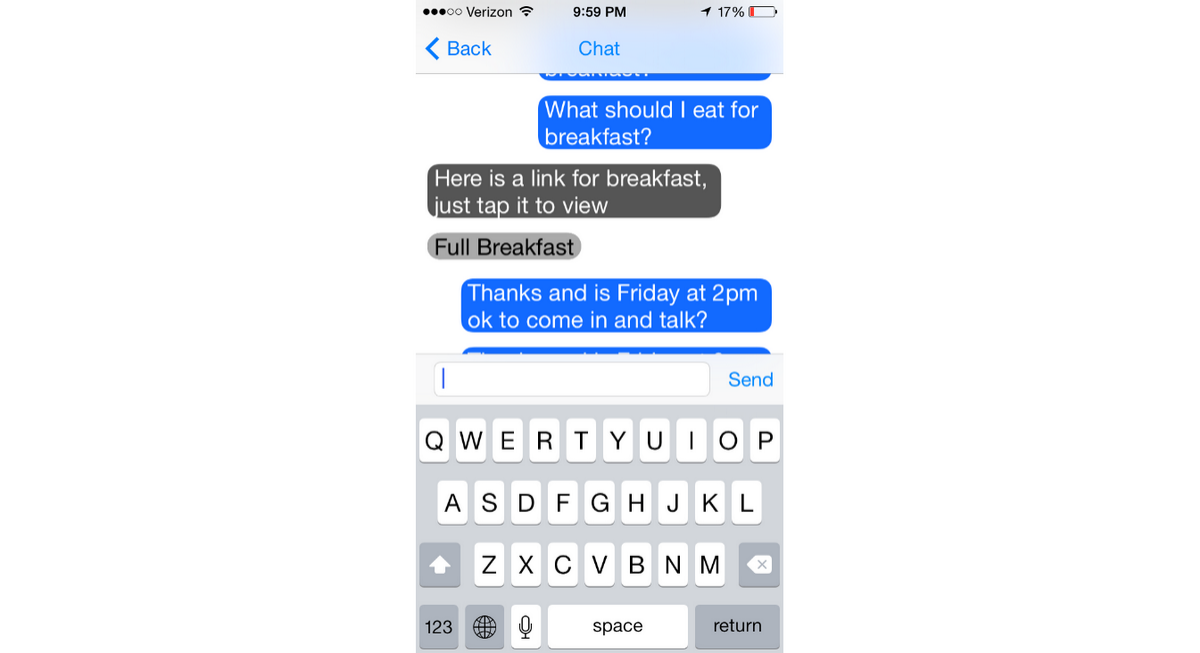
The Chat window, where participants can communicate with their coach.

We have previously described the development of DASH Mobile [9]. The intervention consisted of a smartphone app to track diet, blood pressure, weight, and physical activity daily, combined with a human coach. During the introductory training session with the DASH health coach, participants were taught how to use the app. The home screen of the app defaults to the current day, where participants self-tracked their diet using a novel, simplified data entry tool based on the DASH portions-based approach to dietary intake (Figure 1). At the bottom of the screen, participants could navigate to any of several tabs: Progress, where they could view all tracked behavior, including diet, blood pressure, weight, and steps; Goal, where editable active and achieved goals were listed (Figure 2); Chat, where participants could communicate with their coach (Figure 3); and Education, which linked to DASH-related resources.

Participants were instructed to track other data several times a day via the wireless devices provided. Bluetooth-enabled wireless sensors (iHealth Labs) from these devices transmitted data for blood pressure, weight, and physical activity to the app. Syncing of data occurred manually when the app was opened; data were uploaded to a web-based coaching portal for patients and the health coach to view the participants’ progress. All information transmitted between the phone app and the server was encrypted.

The coach was a master’s student trained to use motivational interviewing as a counseling framework to address behavioral health change. Over the course of 13 weeks, the coach interacted with study participants on a weekly basis through a structured, yet flexible, program that consisted of both telephone calls and asynchronous sessions through the communication channel of their preference (instant message, SMS, or email). In addition, over the course of the study, the DASH health coach logged dietary and physical activity data based on assessments made during 6 phone calls during the study period. Based on this data, the DASH coach assisted participants in setting goals for changing behaviors consistent with their hypertension behavior change plan by reviewing collected data with the user, assigning educational tasks, and engaging in problem solving.

### Outcomes

#### Engagement and Acceptability

To assess participant acceptability of and engagement with the program, the number of blood pressure measurements, weight measurements, and daily steps were logged. Daily step counts of ≤250 were considered invalid. The number of coaching phone calls attempted and completed, servings documented in the dietary assessment, and goals set were also assessed.

#### Physiological Parameters

Blood pressure, heart rate, weight, and steps were collected to assess changes pre- and postintervention. Wireless behavior-tracking devices (blood pressure cuff, scale, and pedometer) were synced via Bluetooth to the mobile app on the participant’s smartphone, which uploaded data to the web-based coaching portal.

### Statistical Analysis

Descriptive analyses were performed to summarize the characteristics of our study population. A Wilcoxon signed rank test comparing the first and last documented physiologic measures (blood pressure, heart rate, weight, body mass index, and calories) was performed to assess significant changes post-intervention. The same method was applied to examine changes between the baseline (days 1-7) and follow-up (days 46-120) for the average daily steps. For all tests, *P* values <.05 were considered statistically significant. Analysis was conducted using SAS 9.4 (SAS Institute).

## Results

### Overview

A total of 17 patients participated in this feasibility pilot study. The mean age of the participants was 59 years (SD 6), and 10/17 (60%) were women. The participants in the study had an average baseline BMI of 33.6 (SD 7.46), an average baseline systolic blood pressure of 138.6 (SD 21.47), and average baseline diastolic blood pressure of 86.9 (SD 16.10).

### Engagement and Acceptability

As detailed in Table 1, participants were engaged with the app. All participants logged their weight, steps, and dietary intake and received messages from the coach. Most of the participants (15/17, 88%) utilized the chat feature to send messages to the coach, and 16 participants (94%) logged their blood pressure and recorded goals. Over 120 days, the mean number of logged measurements per patient was 63 (SD 46) for blood pressure, 52 (SD 45) for weight, and 55 (SD 36) for step counts. Of 102 coaching phone calls, 74 (73%) were completed. The mean number of food entries per day per patient was 5.9 (SD 4.5), and patients set a mean number of 5 (SD 2) goals.

**Table 1:** Engagement measures.

Engagement measures^a^	Engagement over study duration^b^			Engagement per day, mean (SD)
	Mean (SD)	Median (IQR)		
Messages sent to the coach per person	19.3 (16.2)	14 (66)	0.16 (0.14)	
Messages sent from the coach per person	31.6 (16.1)	31 (74)	0.26 (0.13)	
Number of times blood pressure was logged	63.1 (46.0)	69 (178)	0.52 (0.38)	
Number of times weight was logged	51.5 (45.1)	41 (76)	0.43 (0.38)	
Number of times steps were logged	55.1 (35.7)	63 (103)	0.46 (0.30)	
Logged food entries	708.9 (541.2)	616 (1473)	5.9 (4.5)	
Goals recorded	5.1 (1.8)	5.5 (5)	N/A^c^	

^a^Data represented reflect those who used the respective features.

^b^The study duration was 120 days.

^c^N/A: not applicable.

### Physiological Results

Mean systolic and diastolic blood pressure, heart rate, weight, body mass index, calories, and step counts did not change significantly over time (Multimedia Appendix 1).

## Discussion

### Overview

In this pilot study, we found that an interactive mobile app (DASH Mobile) to promote healthy behaviors that reduce hypertension was feasible and engaged participants. We did not find a difference in physiological outcomes, but we were underpowered to identify such changes.

The average participant routinely logged their blood pressure, weight, steps, food servings and communicated with their DASH Mobile health coach, demonstrating active participation. The engagement findings compare favorably with outcomes for the original web-based implementation of DASH (DASH for Health), which reported only 26% participation by the end of the 12-month trial in 2008 [5]. More recently, our findings were similar to another web-based implementation of DASH that reported 71% participation [10].

Mobile interventions are more successful with participant adherence [6,11,12]. Adherence promotion can be designed through several approaches including participant-tailored content, providing real time feedback to the participant, and automated or human support to reinforce the behavior change potential of the digital intervention [13-15]. Combining these approaches may increase the effectiveness of an intervention [16]. For example, a recent weight loss study that compared a solely mobile intervention to a mobile intervention blended with in-person components demonstrated that participants in the blended model experienced a greater average weight loss than those who received a purely mobile intervention [16].

We also identified challenges to engagement. Although the Bluetooth-enabled devices were meant to make the experience of collecting physiologic and behavioral data seamless, we experienced difficulties in pairing the devices with the app, resulting in the need to transition to another company’s devices. Additionally, scheduling synchronous telephone counseling presented a challenge when the coach’s availability did not mesh well with participants’ schedules. Like other behavior change programs, motivating participants to read the educational materials remained a challenge. A potential opportunity for improvement could be to tie these closer to the user-facing data visualizations. Finally, while we used line graphs to illustrate data over time, further work should explore patient preferences.

Given the level of engagement in our study, particularly in the area of tracking, a mobile app intervention may be an effective way to integrate and sustain behavior change in hypertension management. Our study did not have enough participants to determine a change in clinical outcome. A power calculation indicates that at least 140 participants would be needed to evaluate the effectiveness of DASH Mobile. Our favorable engagement findings support such a trial. Successful lifestyle change interventions require substantial patient motivation, engagement, and access to experts.

### Limitations

This study is limited in its generalizability, as all participants were required to be English speaking and own a smartphone. Though 77% of all Americans own a smartphone, only 46% of Americans aged 65 years or older do [8]. In addition, all measurements were self-administered and thus subject to inaccuracy and bias. Furthermore, the intervention is not autonomous, as it included human-based coaching. However, the incorporation of human-based support is a common feature of effective behavior change programs and does not necessarily preclude dissemination [17-19].

In 2017, the American Heart Association/American College of Cardiology published updated hypertension guidelines [20]. Stage 1 hypertension is now defined as a systolic blood pressure of 130-139 mm Hg or a diastolic blood pressure of 80-89 mm Hg, and Stage 2 hypertension is defined as a systolic blood pressure ≥140 mm Hg or a diastolic blood pressure ≥90 mm Hg. However, treatment for Stage 1 and Stage 2 hypertension still include the lifestyle changes promoted by DASH.

### Conclusion

This pilot study demonstrates the feasibility of delivering a digital DASH intervention by leveraging off-the-shelf wireless devices. Participants were engaged, suggesting that a smartphone-based app can be used to deliver behavioral interventions. Future implementations might employ integrated calendars, alarms, social networks, and other smartphone tools to further enhance patient engagement and ultimately clinical outcomes. In summary, our data support the growing interest in using mobile platforms to strengthen user engagement and the accessibility of health behavior change interventions.

## Appendix

Multimedia Appendix 1 Change in physiological outcomes from baseline to follow-up.

## Abbreviations

DASH: Dietary Approaches to Stopping Hypertension
