# Supplementary material for: A Mobile Health Coaching Intervention for Controlling Hypertension: Single-Arm Pilot Pre-Post Study
Source: JMIR Form Res. 2020 May 7;4(5):e13989. doi: 10.2196/13989 (PMC7243130; doi:10.2196/13989)
Supplement: Multimedia Appendix 1 [file formative_v4i5e13989_app1.docx]

Supplemental Table 1: Change in Physiological Outcomes from Baseline to Follow up

| Outcome | Baseline | Last documented | Change | P value |
| --- | --- | --- | --- | --- |
|  | **Mean (SD^a^)** | | |  |
| Blood pressure (n=16) |  |  |  |  |
| Systolic blood pressure | 138.56 (21.47) | 139.75 (15.85) | 1.19 (29.65) | .99 |
| Diastolic blood pressure | 86.88 (16.10) | 89.50 (13.85) | 2.63 (23.64) | .79 |
| Heart rate (n=16) | 79.69 (15.12) | 78.00 (12.55) | -1.69 (13.18) | .72 |
| Weight (n=16) | 212.70 (46.86) | 214.16 (49.52) | 1.47 (9.48) | .75 |
| Body Mass Index (n=16) | 33.61 (7.46) | 33.83 (7.64) | 0.22 (1.40) | .89 |
| Calories (n=17) | 1761.7 (421.51) | 1494.2 (608.03) | -267.50 (526.1) | .11 |
| Steps | 5070.3 (2292.8)^b^ | 5802.4 (2032.8)^c^ | 732.0 (1709.3) | .22 |

^a^ Standard Deviation

^b^ Days 1-7

^c^ Days 46 - 120
